# Psychometric properties and reliability of the Assessment Screen to Identify Survivors Toolkit for Gender Based Violence (ASIST-GBV): results from humanitarian settings in Ethiopia and Colombia

**DOI:** 10.1186/s13031-016-0068-7

**Published:** 2016-02-09

**Authors:** Alexander Vu, Andrea Wirtz, Kiemanh Pham, Sonal Singh, Leonard Rubenstein, Nancy Glass, Nancy Perrin

**Affiliations:** Department of Emergency Medicine, Johns Hopkins University, School of Medicine, Baltimore, USA; Department of International Health, Center for Refugee and Disaster Response, Johns Hopkins University, Bloomberg School of Public Health, Baltimore, USA; Department of Epidemiology, Center for Public Health and Human Rights, Johns Hopkins University, Bloomberg School of Public Health, Baltimore, USA; Johns Hopkins University, School of Nursing , Baltimore, USA; Department of Medicine, Johns Hopkins University, Johns Hopkins Medical Institutions, Baltimore, USA; Center for Health Research Kaiser Permanente Northwest, Portland, OR USA

**Keywords:** Gender-based violence, Screening, Conflict, Refugees, Internally-displaced person, Humanitarian setting, Ethiopia, Colombia, Psychometric analysis

## Abstract

**Background:**

Refugees and internally displaced persons who are affected by armed-conflict are at increased vulnerability to some forms of sexual violence or other types of gender-based violence. A validated, brief and easy-to-administer screening tool will help service providers identify GBV survivors and refer them to appropriate GBV services. To date, no such GBV screening tool exists. We developed the 7-item ASIST-GBV screening tool from qualitative research that included individual interviews and focus groups with GBV refugee and IDP survivors. This study presents the psychometric properties of the ASIST-GBV with female refugees living in Ethiopia and IDPs in Colombia.

**Methods:**

Several strategies were used to validate ASIST-GBV, including a 3 month implementation to validate the brief screening tool with women/girls seeking health services, aged ≥15 years in Ethiopia (*N* = 487) and female IDPs aged ≥ 18 years in Colombia (*N* = 511).

**Results:**

High proportions of women screened positive for past-year GBV according to the ASIST-GBV: 50.6 % in Ethiopia and 63.4 % in Colombia. The factor analysis identified a single dimension, meaning that all items loaded on the single factor. Cronbach’s α = 0.77. A 2-parameter logistic IRT model was used for estimating the precision and discriminating power of each item. Item difficulty varied across the continuum of GBV experiences in the following order (lowest to highest): threats of violence (0.690), physical violence (1.28), forced sex (2.49), coercive sex for survival (2.25), forced marriage (3.51), and forced pregnancy (6.33). Discrimination results showed that forced pregnancy was the item with the strongest ability to discriminate between different levels of GBV. Physical violence and forced sex also have higher levels of discrimination with threats of violence discriminating among women at the low end of the GBV continuum and coercive sex for survival among women at the mid-range of the continuum.

**Conclusion:**

The findings demonstrate that the ASIST-GBV has strong psychometric properties and good reliability. The tool can be used to screen and identify female GBV survivors confidentially and efficiently among IDPs in Colombia and refugees in Ethiopia. Early identification of GBV survivors can enable safety planning, early referral for treatment, and psychosocial support to prevent long-term harmful consequence of GBV.

## Background

Refugees and internally displaced persons (IDPs) who are affected by armed-conflict are at increased vulnerability to some forms of sexual violence or other types of gender-based violence (GBV) [1–4]. GBV is defined in the United Nations Declaration as any act “that results in… physical, sexual or psychological harm or suffering to a person, including threats of such acts, coercion or arbitrary deprivations of liberty, whether occurring in public or in private life…and should encompass, but not be limited to, acts of physical, sexual, and psychological violence in the family, community, or perpetrated or condoned by the State, wherever it occurs.” [5] GBV causes a range of serious immediate and long-lasting physical, [2, 6] reproductive, [7, 8] and psychological harm [2]. A recent systematic review and meta-analysis showed that the prevalence of sexual violence among conflict-affected female refugees and IDPs was 21.4 %, [9] a figure that was likely an underestimation of the true prevalence of sexual violence [10, 11].

Despite the enormity of the problem, the global burden of GBV in conflict and post-conflict settings remains elusive and is of great concern to all frontline actors. Moreover, while services specific to GBV are available in many humanitarian settings, under-reporting and underuse of these services by GBV survivors is common. A validated, brief and easy-to-administer screening tool will help service providers identify GBV survivors and refer them to appropriate GBV services. Currently, no such screening tool exists. The overall objective of the study was to increase disclosure and access to existing services for GBV survivors. We undertook a systematic process to develop a screening tool, known as the Assessment Screen to Identify Survivors Toolkit for Gender Based Violence (ASIST-GBV), to confidentially identify GBV survivors among refugees and internally displace persons (IDPs) who were affected by armed-conflict [12–14]. The objective of this analysis was to assess the psychometric properties of the ASIST-GBV screening instrument when administered among refugees living in Ethiopia and IDPs in Colombia.

## Methods

The overall study was conducted in 2011 through 2013 in Ethiopia and Colombia. Sites were selected during collaborative discussion with UNHCR. Ethiopia was selected as a study site because it currently provides support close to 730,000 refugees, most of whom are from Burundi, Democratic Republic of Congo, Eritrea, Rwanda, Somalia and South Sudan [15]. Colombia was selected as a study site because it has one of the largest population of IDPs globally, an estimated 5.8 million IDPs living within the country as of December 2014 [16]. The development of the screening tool involves the following processes: A) conducting a *systematic review* to identify any existing screening tools that have been used to identify GBV. B) *Qualitative research* focused on domains relevant to the screening tool to describe the various types, locations and perpetrators of GBV; explore the current barriers to survivors’ reporting and service seeking behaviors; and explore service providers’ barriers to proving care to survivors and obtain suggestions for the development of the screening tool [12, 13]. The results of the qualitative work provided the foundation to design the ASIST-GBV screening questionnaire. C) A *piloting phase* was conducted after the development of the ASIST-GBV screening tool. These methods have been described in greater detail in another publication [14]. D) Finally, an *implementation phase* to assess the performance of the tool among a population that has not been exposed to the development of the screening tool.

This study presents the results of the implementation phase of the ASIST-GBV applied among the general populations of refugee/IDP women seeking health services in Ethiopia and Colombia. Table 1 provides the items of the ASIST-GBV. There was one item, forced abortion, on the Colombia version of the ASIST-GBV that was not included in Ethiopia because qualitative findings noted this type of violence was prevalent in Colombia and the item was added later after implementation was completed in Ethiopia. Therefore, the psychometric analysis focused on the 6 common items on the two versions of the ASIST-GBV.

**Table 1:** ASIST-GBV screening instrument

GBV Screening Question Items:
1. In the past year, have you been threatened with physical or sexual violence by someone in your home or outside of your home?
2. In the past year, have you been hit, punched, kicked, slapped, choked, hurt with a weapon, or otherwise physically hurt by someone in your home or outside of your house?
3. In the past year, were you forced to have sex against your will?
4. In the past year, were you forced to have sex to be able to eat, have shelter, or have sex for essential services (such as protection or school) because you or someone in your family would be in physical danger if you refused?
5. In the past year, were you physically forced or made to feel that you had to become pregnant against your will?
6. In the past year, were you coerced or forced into marriage?
7. In the past year, were you coerced or forced to have an abortion?
*If yes to any of items 1 to 7, the woman has screened positive for gender-based violence. If positive screen, please ask:*
*8.* Would you like to talk to someone or learn more about services for women who have experienced gender-based violence?

### Settings and participants

In Ethiopia, data collection for this validation took place during 3-month implementation at health facilities in the Bokolomayo refugee camp, within the Dolo Ado area, from June through August 2012. The population of Bokolomayo refugee camp was predominantly Somoli refugees due to acute humanitarian famine crisis in Somolia in 2012 [17]. In Colombia, data collection was completed in a hospital setting in Mocoa during February through May 2013. All women were privately offered screening with the ASIST-GBV, as part of routine health center and hospital services. A total of 998 participants were enrolled into the study (*N* = 511 IDPs in Colombia and *N* = 487 refugees living in Ethiopia). Inclusion criteria for participants were female, aged 15 years and older in Ethiopia and 18 years and older in Colombia, and self-reported status as refugee (in Ethiopia) or internally displaced (in Colombia). Women judged, in collaboration with local partner organizations, to be cognitively impaired or too traumatized to participate were excluded from the study.

### Human subjects protection

Research and ethical approvals were obtained from local ministries (Colombian Ministry of Social Protection and the Ethiopian governmental agency responsible for all refugee related affairs, Administration for Refugee and Returnee Affairs (ARRA)) and from Johns Hopkins Medical Institutes Institutional Review Board (IRB No.: NA_00049747 for Colombia and NA_00042672 for Ethiopia). The inclusion of participants aged 15 years or older in the study was to assess the vulnerability of young women and girls to GBV. However, most ethical committees require parental consent from candidate participants under 18 years of age. As it may be possible that some parents may perpetrate GBV or may stigmatize survivors, we requested a waiver of parental consent. The waiver was only granted in Ethiopia and not in Colombia. Thus, to protect participants’ confidentiality, the decision was made to include only participants aged 18 years of age and older in Colombia.

In Ethiopia, the consent forms were developed in consultation with local non-governmental organizations providing GBV and child protection services to adult and child refugees. GBV and child protection service providers (PAPDA and Save the Children) offered services to all age ranges in Bokolomayo. The training to all providers who conducted screening included specific training that addressed confidentiality, safety (abuse/violence perpetrated by parent/family member) and health needs of participants ages 15–17 years. The training and implementation process included the referral pathways established with local GBV and child protection programs and services. Interviewers are trained to make sure that all participants regardless of age have the option to stop or withdraw from the screening process at any time that they wish and by doing so there would not be any negative consequences on the participant or their family. It is important to note that although screening participants could be ages 15–17 years, they also could be married and mothers at this age and therefore it is important not to assume that age alone would dictate referrals to child protection or specific services for children or adolescents. Participants who were identified with recent GBV were referred into an established GBVIMS intake procedure in Bokolomayo regardless of age, and referral services included child protection. The GBV and child protection programs were in place prior to study and partners in study implementation.

### Recruitment and consent

Female refugees/IDPs who were attending the local health clinics were privately invited to be screened by trained social workers (Ethiopia) and nurses (Colombia). The screening questionnaire was professionally translated into Somali (Ethiopia) and Spanish (Colombia). Cognitive testing of the translated Somali and Spanish versions of the screening tool was done to minimize potential measurement and response errors of the screening tool. Five Somali female refugee survivors in Ethiopia and five IDP female survivors in Colombia participated in the cognitive testing. Participants in both countries were survivors who were recruited from existing GBV programs. The tool was then back-translated to confirm the language was correct. Edits were made through an iterative process to ensure that the translations captured the intended meaning of the statements. Pilot testing of ASIST-GBV was conducted in each country. Participants were informed of the project, including the purpose, risks, benefits and study safety procedures to ensure protection of research participants. No personal identifying data was collected. No incentives were given to participants. The ability of clients to receive services were not affected if client accepted or refused to undergo the screening. Because written consents would enable potentials of linkage of participant’s name to the study, verbal consents were used to ensure participants’ confidentiality and safety. All participants were provided GBV information, services and resources at the completion of the screening questionnaire, regardless of results. Participants who screened positive to recent experiences of GBV were offerred referral to the local implementing partners, the Partnership for Pastoralists Development Agency (PAPDA) or Save the Children social workers who provided case management and psychosocial services (Ethiopia) or directly referral to hospital social workers who provided case management (Colombia). Participant confidentiality was strictly enforced per IRB protocols [14].

### Statistical analysis

Each of the ASIST-GBV items was scored 0 or 1 (no/yes) (Table 1). Women screened positive for GBV if they responded “yes” to any one or more of the items. To examine the psychometric properties of the ASIST-GBV, factor analysis, Cronbach’s α, and item response theory (IRT) models were conducted. IRT examines the relationship between a participant’s position on a latent trait (in this case degree of experience of GBV) and the probability that they disclosed experience on the different items of the ASIST-GBV. IRT places items on a common hierarchy with items higher on the hierarchy associated with a stronger degree of the trait (experiencing a higher degree of GBV). This approach is particularly useful for binary responses where an event did or did not happen. For a screening tool, IRT as an approach to establishing the psychometric property of the instrument is more appropriate than classic test theory which assumes that all items on instrument can be used as parallel measures of the degree of the latent trait [18]. While the formative work in Colombia identified an additional item, forced abortion, to the 6 common item screening questionnaire, the psychometric analysis focused on the 6 common items used across the two countries. A 2-parameter IRT model was used [18]. The first parameter estimated for each item “difficulty” or the probability of endorsing an item given varying levels of the latent trait. The second parameter estimated “discrimination” for each item or the ability of that item to discriminate among people with various levels of the latent trait. We also tested for differential item functioning (DIF) across countries. This allowed us to determine if the measurement characteristics of ASIST-GBV were the same for the two countries. One assumption of the IRT model is that the items on the ASIST-GBV tool were unidimensional. This can be examined with factor analysis. Exploratory factor analysis using principle components was conducted to assess if the 6-items were unidimensional. Cronbach α reliabilities were computed to assess the internal consistency of the construct.

## Results

Table 2 shows the participant characteristics per site. High proportions of women screened positive for past year GBV according to the ASIST-GBV: 50.6 % in Ethiopia and 63.4 % in Colombia (Colombia: *n* = 319/511; Ethiopia: *n* = 244/487; Table 3). Types of GBV identified on the ASIST-GBV in Ethiopia included: threats of violence (35.7 %), physical violence (46.6 %), forced sex (20.4 %), coercive sex for survival (27.7 %), forced pregnancy (15.8 %), and forced marriage (19.9 %). In Colombia, participants reported threats of violence (41.5 %), physical violence (23.5 %), forced sex (36.0 %), coercive sex for survival (20.2 %), forced pregnancy (1.98 %), forced marriage (4.17 %), and forced abortion (1.59 %). It is important to note that the proportions of the different types of GBV for this study should not be interpreted as prevalence data because the method used in this study was not designed to be implemented as a population based sampling.

**Table 2:** Sociodemographic characteristics of female refugee and internally displaced participants in Ethiopia and Colombia, respectively (*N* = 998)

	Country				Total	
	Colombia (*N* = 511)		Ethiopia (*N* = 487)		(*N* = 998)	
Characteristics	n	col %	n	col %	n	col %
Median age (range)	30	(18–62)	29	(15–81)	29	15–81
Age distributions						
15–17	0	0	23	4.7	23	2.3
18–24	138	27	113	23.2	251	25.2
25–34	207	40.5	221	45.4	428	42.9
35–44	143	28	106	21.8	249	24.9
45–59	19	3.7	16	3.3	35	3.5
> = 60	4	0.8	8	1.6	12	1.2
Colombian Ethnicity						
Mestizo/Blanco	389	76.9	N/A	N/A	389	76.9
Afro Desceniente	43	8.5	N/A	N/A	43	8.5
Indigena	72	14.2	N/A	N/A	72	14.2
Raizal de Archipelago	1	0.2	N/A	N/A	1	0.2
Other	1	0.2	N/A	N/A	1	0.2
Refugee Country of Origin						
Somalia	N/A	N/A	480	100	480	100
Median Years Displaced (range)	7	(0–15)	2	(0–20)	3	0–20
Distribution of Time Displaced						
Less than 2 yrs	4	0.78	56	11.5	60	6.01
2–3 yrs.	32	6.26	408	83.78	440	44.09
4–7 yrs.	238	46.58	11	2.26	249	24.95
More than 7	237	46.38	12	2.46	249	24.95
Marital Status						
Single	156	30.8	37	7.8	193	19.6
Married/Living together	291	57.4	401	84.1	692	70.3
Formerly married	60	11.8	39	8.2	99	10.1
Education Completed						
Never	13	2.6	249	56.7	262	28
Pre-school or Primary	198	39.9	136	31	334	35.7
Secondary	244	49.2	51	11.6	295	31.6
Technical	41	8.3	0	0	41	4.4
University or higher	0	0	3	0.7	3	0.3

**Table 3:** Distribution of responses to ASIST-GBV screening items, by country (*N* = 998)

ASIST-GBV Questions (last 12mo.)	Country				Total	
	Colombia (*N* = 511)		Ethiopia (*N* = 487)		Total (*N* = 998)	
	n	col %	n	col %	n	col %
Threatened with physical or sexual violence (*n* = 988)						
No	296	58.5	310	64.3	606	61.3
Yes	210	41.5	172	35.7	382	38.7
Hit, punched, kicked, slapped, choked, hurt with a weapon or otherwise physically hurt (*n* = 986)						
No	386	76.3	257	53.4	643	65.1
Yes	119	23.5	224	46.6	343	34.8
Forced to have sex against will (*n* = 986)						
No	324	64	382	79.6	706	71.6
Yes	182	36	98	20.4	280	28.4
Forced to have sex to be able to eat, have shelter, protect family, or for essential service (*n* = 985)						
No	403	79.8	347	72.3	750	76.1
Yes	102	20.2	133	27.7	235	23.9
Physically forced or made to feel she had to become pregnant (*n* = 985)						
No	495	98	404	84.2	899	91.3
Yes	10	2	76	15.8	86	8.7
Forced to end a pregnancy by physical violence, medication, or to seek clinic services (*n* = 503)^a^						
No	495	98.2	N/A	N/A	495	98.2
Yes	8	1.6	N/A	N/A	8	1.6
Coerced or forced into marriage (*n* = 982)						
No	483	95.8	383	80.1	866	88.2
Yes	21	4.2	95	19.9	116	11.8
Positive by ASIST-GBV Screen (*n* = 985)						
No	184	36.6	238	49.4	422	42.8
Yes	319	63.4	244	50.6	563	57.2

^a^ Item addressing forced termination of pregnancy included later during Colombia study and was not included in early tool implemented in Ethiopia

### Psychometric properties of ASIST-GBV

**Fig. 1:**
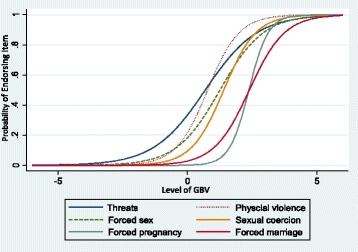
Item Response Curves from the 2-parameter model

The factor analysis identified single dimension (1^st^ 3 Eigenvalues: 2.84, 0.94, 0.82) that accounted for 47 % of the variance. All items loaded on the single factor (factor loadings: threat of violence 0.51, physical violence 0.65, forced sex 0.58, coercive sex for survival 0.69, forced pregnancy 0.63, and forced marriage 0.57). Cronbach’s α was at 0.77, in which the traditional threshold of acceptable internal consistency or reliability of α coefficient being equal to 0.70 or greater [19]. A 2-parameter logistic IRT model was used for estimating the precision and discriminating power of each item. Table 4 presents the item difficulty and discrimination for each of the items. Figure 1 graphs the corresponding item functioning curves. As the curves move to the right along the x-axis, item difficulty increases; as the slope of the curve becomes steeper, the discrimination of the item (ability to detect differences in the degree of the latent trait) increases. Item difficulty illustrated the hierarchy of the items. Item difficulty varied across the continuum of GBV experiences in the following order (lowest to highest): threats of violence (.690), physical violence (1.28), forced sex (1.49), coercive sex for survival (2.25), forced marriage (3.51), and forced pregnancy (6.33). Women reporting few experiences of GBV were more likely to report threats of violence but not the other items on the tool. Whereas, women who reported experiencing more GBV were more likely to report physical violence and forced sex, in addition to threats of violence. Women who reported experiencing the greatest amount of GBV were likely to report all of the items. Discrimination results showed that forced pregnancy was the item with the strongest ability to discriminate between different levels of GBV. Because it had a high difficulty, it was discriminating women at the highest end of the GBV continuum, or women that experience the most GBV. Physical violence and forced sex also had higher levels of discrimination with threats of violence discriminating among women at the low end of the GBV continuum and coercive sex for survival among women at the mid-range of the continuum.

**Table 4:** Item difficulty and discrimination from the 2-PL model

Item	Difficulty	Discrimination
Threats of violence	.690	1
Physical violence	1.279	1.64
Forced Sex	1.493	1.19
Sexual coercion	2.254	1.61
Forced Marriage	3.509	1.47
Forced Pregnancy	6.331	2.67

DIF was found for two of the six items on the scale: physical and forced sex. This implied that reporting of these items was associated with differing degrees of GBV in Ethiopia and Colombia. Physical violence was commonly reported by women who experienced two or more types of GBV in Colombia whereas it was reported by women who experienced one or more types of GBV in Ethiopia. Forced sex was commonly reported by women who experienced one or more types of GBV in Colombia where it is reported by women who experienced three or more types of GBV items in Ethiopia. Forced sex was more common in Colombia (36 %) than Ethiopia (20 %); the reverse pattern was seen for physical violence, with Colombia at 23 % and Ethiopia at 46 %.

## Discussion

The ASIST-GBV screening instrument was developed using comprehensive methods to build the body of evidence in support of the application of the screening tool to confidentially and efficiently administer the tool, to identify women early and refer them to essential GBV services in humanitarian settings.

In this study, the findings demonstrated that the ASIST-GBV instrument had strong psychometric properties and good reliability. The item difficulty and discrimination results illustrated that the six ASIST-GBV items common across the Ethiopia and Colombia versions captured the different levels and types of GBV experiences. Importantly, the items were able to discriminate across the continuum of GBV experienced by women, with threat of violence discriminating among women at the low end of the scale; physical violence and forced sex in the mid-range of the continuum; and forced pregnancy discriminating among women at the high end of the scale. The GBV items were unidimensional and all items loaded onto a single factor. The internal consistency of the tool is adequate.

The ASIST-GBV screening instrument is perhaps the first tool of its kind that was designed and validated to safely identify past year GBV in a humanitarian settings. It is also the first tool, to our knowledge, to screen more broadly for GBV, rather than intimate partner violence (IPV). The primary intent of the GBV screening tool was to *identify survivors with recent, untreated experiences of GBV for referral*. The questionnaire does not attempt to document an exhaustive list of all the different type of violence women/girls experience. Rather, the screening tool focused on the types of GBV where services are available such as health care treatment and psychosocial support. The brevity and ease of use of the tool were designed to address issues of limited staffing capacity and limited time that service providers have when working in challenging humanitarian emergencies. The ASIST-GBV complements current existing comprehensive GBV assessment tools as well as existing services because survivors identified through the screening process of the ASIST-GBV can be linked to GBV services.

Beyond the development and demonstration of good psychometric properties and reliability of the ASIST-GBV instrument, the tool is being used in partnership with various non-governmental organizations to implement among refugees in Lebanon, Kenya and Uganda. We have also expanded the use of the screening tool to the general population in Somalia and developed a tool appropriate for male survivors of GBV in partnership with colleagues in Uganda [20]. The collective experiences among the service providers across various service sectors who have used the ASIST-GBV in the past two years have reported the feasibility of administration in less than five minutes, allowing service providers to incorporate the screening as part of programming and daily activity with minimal increase in work responsibilities. Additionally, the tool enables providers involved in GBV services to further their work by proactively identifying survivors earlier compared to traditional, passive practices. Finally, the discriminate properties allows providers to tailor referrals and linkages of service to appropriate individual women’s health and social support needs.

There were several potential limitations to the study. We collected data only on participants who gave consent and did not collect data on the number of females who refused screening or why participants refused screening. The ASIST-GBV does not capture the full experience and all forms of GBV [5]. The forms of violence that were included in the screening tool were developed based on the qualitative research that informed the development of a GBV screening tool that enable early identification of survivors in order to link them to existing services [12, 13]. The tool took into account the facilitators and barriers to accessing health services to address these needs from the perspectives of the refugee respondents and GBV service providers. This focus on identification of GBV that could be addressed by the health system and would not overwhelm staff capacity. Thus, the tool was limited to experiences within the last 12 months and excluded such forms of GBV, such as female genital mutilation, which would overwhelm the health system and for which multiple interventions are already present in the targeted settings. While the screening tool creates an opportunity for respondents to participate in screening, the tool cannot account for the various factors and complex challenges involved in the respondent’s willingness to disclose one’s GBV experience. These barriers to reporting GBV experiences are multi-factorial and may include on-going violence in the domestic setting, financial dependence on spouse, safety of one’s children, trust in the local police and judicial system. It is possible that participants who have experienced recent GBV may decided not to disclose the violence related to existing barriers. Nonetheless, through screening, there is increased awareness among the community and survivors that services are available and healthcare workers are interested in providing assistance. As there is no existing GBV screening tool, we were not able to assess the criterion validity of the ASIST-GBV instrument against a previously developed tool. The test and re-test approach could not be performed because our ethical protocols did not allow us to collect personal identifiers to follow-up with clients. While the DIF analysis was acceptable when comparing the performance of the tool in Ethiopia and Colombia, we do not know how well the tool will perform when applied in different countries, different cultures and different humanitarian and development contexts. However, as noted above, we are working with partner organizations in diverse countries to answer questions related to the generalizability of the ASIST-GBV tool.

## Conclusion

The ASIST-GBV is a 7-item screening tool that was developed using a rigorous mixed-methods approach and validated in various displaced populations and settings. The IRT analyses that were performed on the six common items across the two countries suggested that the ASIST-GBV screening tool had sufficient discriminating power for each item and can assess GBV across a continuum. The tool can be used to screen and identify female GBV survivors confidentially and efficiently among IDPs in Colombia and refugees in Ethiopia. Early identification of GBV survivors can enable early referral for treatment and psychosocial support to prevent long-term harmful consequence of GBV.

## Abbreviations

ARRA: Administration for Refugee and Returnee Affairs
ASIST-GBV: assessment screen to identify survivors toolkit for gender based violence
DIF: differential item functioning
GBV: gender-based violence
GBV-IMS: gender based violence information management system
IDP: internally displaced persons
IRB: Institutional Review Board
IRT: item response theory
PAPDA: Partnership for Pastoralists Development Agency
UNHCR: United Nations High Commissioner for Refugees
